# An observational prospective cohort study of naloxone use at witnessed overdoses, Kazakhstan, Kyrgyzstan, Tajikistan, Ukraine

**DOI:** 10.2471/BLT.21.286459

**Published:** 2022-02-03

**Authors:** Paul Dietze, Gilberto Gerra, Vladimir Poznyak, Giovanna Campello, Wataru Kashino, Dzhonbek Dzhonbekov, Tetiana Kiriazova, Danil Nikitin, Assel Terlikbayeva, Dzmitry Krupchanka, Anja Busse

**Affiliations:** a Behaviours and Health Risks Program, Burnet Institute, 85 Commercial Rd, Melbourne, VIC, 3004, Australia.; b Mental Health Department, AUSL of Parma, Parma, Italy.; c Department of Mental Health and Substance Use, World Health Organization, Geneva, Switzerland.; d Drug Prevention and Health Branch, United Nations Office on Drugs and Crime, Vienna, Austria.; e Public Organization Prisma, Dushanbe, Tajikistan.; f Ukrainian Institute on Public Health Policy, Kyiv, Ukraine.; g Global Research Institute Foundation, Bishkek, Kyrgyzstan.; h Global Health Research Center of Central Asia, Columbia School of Social Work, Almaty, Kazakhstan.

## Abstract

**Objective:**

To determine whether participation in the United Nations Office on Drugs and Crime (UNODC) and the World Health Organization’s (WHO) Stop Overdose Safely (S-O-S) take-home naloxone training project in Kazakhstan, Kyrgyzstan, Tajikistan and Ukraine resulted in naloxone use at witnessed opioid overdoses.

**Methods:**

An observational prospective cohort study was performed by recruiting participants in the implementation of the S-O-S project, which was developed as part of the broader S-O-S initiative. Training included instruction on overdose responses and naloxone use. Study participants were followed for 6 months after completing training. The primary study outcome was participants’ naloxone use at witnessed overdoses, reported at follow-up.

**Findings:**

Between 400 and 417 S-O-S project participants were recruited in each country. Overall, 84% (1388/1646) of participants were interviewed at 6-month follow-up. The percentage who reported witnessing an overdose between baseline and follow-up was 20% (71/356) in Tajikistan, 33% (113/349) in Kyrgyzstan, 37% (125/342) in Ukraine and 50% (170/341) in Kazakhstan. The percentage who reported using naloxone at their most recently witnessed overdose was 82% (103/125) in Ukraine, 89% (152/170) in Kazakhstan, 89% (101/113) in Kyrgyzstan and 100% (71/71) in Tajikistan.

**Conclusion:**

Implementation of the UNODC–WHO S-O-S training project in four low- to middle-income countries resulted in the reported use of take-home naloxone at around 90% of witnessed opioid overdoses. The percentage varied between countries but was generally higher than found in previous studies. Take-home naloxone is particularly important in countries where emergency medical responses to opioid overdoses may be limited.

## Résumé

**Objectif:**

Déterminer si la participation au projet de formation aux kits de naloxone à emporter Stop Overdose Safely (S-O-S), lancé conjointement par l'Office des Nations Unies contre les drogues et le crime (ONUDC) et l'Organisation mondiale de la Santé (OMS) au Kazakhstan, au Kirghizistan, au Tadjikistan et en Ukraine, a entraîné l'usage de naloxone lorsqu'une surdose d'opioïdes était constatée.

**Méthodes:**

Nous avons mené une étude de cohorte prospective observationnelle en recrutant des participants à la mise en œuvre du projet S-O-S, développé dans le cadre de la vaste initiative S-O-S. La formation leur expliquait notamment comment réagir face à une overdose et comment utiliser la naloxone. À l'issue de leur formation, les participants à l'étude ont fait l'objet d'un suivi pendant 6 mois. L'étude a essentiellement conclu que les participants ont bien fait usage de naloxone lorsqu'ils ont été confrontés à une overdose, conformément à ce qu'ils ont relaté durant la période de suivi.

**Résultats:**

Entre 400 et 417 participants au projet S-O-S ont été recrutés dans chaque pays. Au total, 84% (1388/1646) d'entre eux ont été interrogés au terme des 6 mois de suivi. Le pourcentage de participants ayant indiqué avoir assisté à une overdose entre la formation initiale et la fin de la période de suivi s'élevait à 20% (71/356) au Tadjikistan, 33% (113/349) au Kirghizistan, 37% (125/342) en Ukraine et 50% (170/341) au Kazakhstan. Enfin, le pourcentage de ceux ayant indiqué avoir eu recours à la naloxone lors de l'overdose la plus récente à laquelle ils ont assisté s'élevait à 82% (103/125) en Ukraine, 89% (152/170) au Kazakhstan, 89% (101/113) au Kirghizistan et 100% (71/71) au Tadjikistan.

**Conclusion:**

Le déploiement du projet de formation S-O-S porté par l'ONUDC et l'OMS dans quatre pays à faible et moyen revenu a encouragé l'usage de kits de naloxone pour 90% des overdoses d'opioïdes constatées. Le pourcentage variait d'un pays à l'autre mais, dans l'ensemble, il était supérieur à celui des études précédentes. Les kits de naloxone à emporter sont particulièrement importants dans les pays où la prise en charge médicale urgente des surdoses d'opioïdes pourrait s'avérer limitée.

## Resumen

**Objetivo:**

Determinar si la participación en el proyecto de formación en naloxona para llevar a casa Stop Overdose Safely (S-O-S) de la Oficina de las Naciones Unidas contra la Droga y el Delito (ONUDD) y la Organización Mundial de la Salud (OMS) en Kazajistán, Kirguistán, Tayikistán y Ucrania generó el uso de naloxona cuando se presenciaron sobredosis de opiáceos.

**Métodos:**

Se realizó un estudio de cohortes prospectivo y observacional mediante el reclutamiento de participantes en la aplicación del proyecto S-O-S, que se desarrolló como parte de la iniciativa más amplia S-O-S. La formación incluía enseñanzas sobre las respuestas a las sobredosis y el uso de la naloxona. Los participantes del estudio fueron seguidos durante 6 meses después de completar la formación. El desenlace primario del estudio fue el uso de naloxona por parte de los participantes ante la presencia de sobredosis, notificado durante el seguimiento.

**Resultados:**

En cada país se reclutaron entre 400 y 417 participantes para el proyecto S-O-S. En general, el 84 % (1388/1646) de los participantes fueron entrevistados en el seguimiento a los 6 meses. El porcentaje que declaró haber presenciado una sobredosis entre el inicio y el seguimiento fue del 20 % (71/356) en Tayikistán, el 33 % (113/349) en Kirguistán, el 37 % (125/342) en Ucrania y el 50 % (170/341) en Kazajistán. El porcentaje que declaró haber utilizado naloxona en las sobredosis presenciadas más recientes fue del 82 % (103/125) en Ucrania, del 89 % (152/170) en Kazajistán, del 89 % (101/113) en Kirguistán y del 100 % (71/71) en Tayikistán.

**Conclusión:**

La ejecución del proyecto de formación S-O-S de la ONUDD y la OMS en cuatro países de ingresos bajos y medios permitió notificar el uso de naloxona para llevar a casa en alrededor del 90 % de las sobredosis de opiáceos presenciadas. El porcentaje varió entre los países, pero en general fue más alto que el encontrado en los estudios anteriores. La naloxona para llevar a casa es de especial importancia en los países donde la respuesta médica de emergencia a las sobredosis de opiáceos podría ser limitada.

## ملخص

**الغرض:**

تحديد ما إذا كانت المشاركة في مكتب الأمم المتحدة الخاص بالمخدرات والجريمة (UNODC)، ومشروع التدريب على إيقاف الجرعة الزائدة من عقار نالوكسون المنزلي بأمان (S-O-S) التابع لمنظمة الصحة العالمية (WHO) في كازاخستان وقيرغيزستان وطاجيكستان وأوكرانيا، قد أدى إلى استخدام عقار نالوكسون في جرعات زائدة مشهودة من الأفيون.

**الطريقة:**

تم إجراء دراسة أترابية قائمة على الملاحظة من خلال تجنيد المشاركين في تنفيذ مشروع S-O-S، الذي تم تطويره كجزء من مبادرة S-O-S الأوسع نطاقًا. وشمل التدريب تعليمات حول الاستجابات للجرعات الزائدة واستخدام عقار نالوكسون. تمت متابعة المشاركين في الدراسة لمدة 6 أشهر بعد إكمال التدريب. كانت نتيجة الدراسة الأولية هي استخدام المشاركين لعقار نالوكسون بجرعات زائدة مشهودة، تم الإبلاغ عنها أثناء المتابعة.

**النتائج:**

تم تجنيد ما بين 400 و417 مشاركًا في مشروع S-O-S في كل دولة. بشكل عام، تمت مقابلة 84% (1388/1646) من المشاركين بشكل شخصي في متابعة استمرت لمدة 6 أشهر. النسبة المئوية للذين أبلغوا عن مشاهدة جرعة زائدة بين خط الأساس والمتابعة كانت 20% (71/356) في طاجيكستان، و33% (113/349) في قيرغيزستان، و37% (125/342) في أوكرانيا، و50% (170/341) في كازاخستان. النسبة المئوية للذين أبلغوا عن استخدام عقار نالوكسون في أحدث جرعة زائدة مشهودة لديهم كانت 82% (103/125) في أوكرانيا، و89% (152/170) في كازاخستان، و89% (101/113) في قيرغيزستان، و100% (71/71) في طاجيكستان.

**الاستنتاج:**

إن تنفيذ مشروع التدريب S-O-S، التابع لمكتب الأمم المتحدة الخاص بالمخدرات والجريمة ومنظمة الصحة العالمية، في أربع دول ذات دخل منخفض إلى دخل متوسط، قد أدى إلى الإبلاغ عن استخدام عقار نالوكسون المنزلي في حوالي 90% من الجرعات الزائدة المشهودة من الأفيون. اختلفت النسبة المئوية بين الدول ولكنها كانت أعلى بشكل عام مما تم العثور عليه في الدراسات السابقة. يعتبر تناول عقار نالوكسون المنزلي مهمًا بشكل خاص في الدول التي قد تكون فيها الاستجابات الطبية الطارئة للجرعات الزائدة من الأفيون محدودة.

## 摘要

**目的:**

旨在确定是否参加联合国毒品和犯罪问题办公室 (UNODC) 和世界卫生组织 (WHO) 在哈萨克斯坦、吉尔吉斯斯坦、塔吉克斯坦和乌克兰开展的安全消除药物过量 (S-O-S) 自主服用纳洛酮培训项目，因为这些国家的鸦片用药过量案例中普遍存在使用纳洛酮的现象。

**方法:**

通过招募参与者参与 S-O-S 项目的实施开展观察性前瞻性队列研究，该 S-O-S 项目是范围更大的 S-O-S 方案的组成部分。培训包括解答药物过量问题和指导纳洛酮的使用方法。完成培训后，会对研究参与者进行为其 6 个月的后续追踪。据后续追踪报道，主要研究结果为参与者表明其见证的用药过量案例中会使用纳洛酮。

**结果:**

在各个国家分别招募了 400 到 417 名 S-O-S 项目参与者。总体来看，对 84% (1388/1646) 参与者进行了为期 6 个月的后续追踪。在基线到后续追踪期间报告存在用药过量的参与者比例为：哈萨克斯坦 50% (170/341)、吉尔吉斯斯坦 33% (113/349)、塔吉克斯坦 20% (71/356) 和乌克兰 37% (125/342)。报告在其最近一次见证的用药过量中使用纳洛酮的参与者比例为：哈萨克斯坦 89% (152/170)、吉尔吉斯斯坦 89% (101/113)、塔吉克斯坦 100% (71/71)、 和乌克兰 82% (103/125)。

**结论:**

在四个中低收入国家实施 UNODC–WHO S-O-S 培训项目后发现，在报告的自主服用纳洛酮案例中，90% 存在鸦片用药过量现象。该比例因不同国家而异，但普遍高于之前研究中发现的数值。在鸦片用药过量应急医疗救治方面资源有限的国家，自主服用纳洛酮的问题尤为严重。

## Резюме

**Цель:**

Определить, привело ли участие в проекте под названием «За безопасную профилактику передозировки» (S-O-S) Управления Организации Объединенных Наций по наркотикам и преступности (УНП ООН) и Всемирной организации здравоохранения (ВОЗ) по обучению использования налоксона в домашних условиях лицами, которые могут стать свидетелями передозировки опиоидов, в Казахстане, Кыргызстане, Таджикистане и на Украине.

**Методы:**

Было проведено обсервационное проспективное когортное исследование путем набора участников в рамках реализации проекта S-O-S, который был разработан на базе более широкой инициативы S-O-S. Обучение состояло из указаний по реагированию на передозировку и использованию налоксона. За участниками исследования наблюдали в течение 6 месяцев после завершения обучения. Главным результатом исследования, о котором сообщалось при последующем наблюдении, было использование налоксона лицами, которые могут стать свидетелями передозировки.

**Результаты:**

В каждой стране было набрано от 400 до 417 участников проекта S-O-S. В целом 84% (1388/1646) участников были опрошены через 6 месяцев наблюдения. Процент тех, кто сообщил о том, что стал свидетелем передозировки, между исходным уровнем и последующим наблюдением составил 20% (71/356) в Таджикистане, 33% (113/349) в Кыргызстане, 37% (125/342) на Украине и 50% (170/341) в Казахстане. Процент лиц, которые стали свидетелями передозировки и использовали налоксон во время последнего такого случая, составил 82% (103/125) на Украине, 89% (152/170) в Казахстане, 89% (101/113) в Кыргызстане и 100% (71/71) в Таджикистане.

**Вывод:**

Реализация проекта S-O-S от УНП ООН и ВОЗ в четырех странах с низким и средним уровнем дохода привела к тому, что примерно в 90% случаев сообщалось об использовании налоксона в домашних условиях лицами, которые стали свидетелями передозировки опиоидов. Процентное значение варьировалось между странами, но в целом было выше, чем в предыдущих исследованиях. Использование налоксона в домашних условиях особенно важно в тех странах, где неотложная медицинская помощь при передозировке опиоидов может быть ограничена.

## Introduction

Opioid overdose is a leading cause of drug-related death.^1^ The risk of an overdose varies with the type of opioid consumed and the population group involved. Longitudinal studies suggest that 2–3% of people who use heroin die each year but higher rates have been observed.^2^^,^^3^ Opioid overdoses are preventable through opioid agonist maintenance treatment, though treatment is not available everywhere and uptake can be low.^4^^,^^5^ Consequently, responses to acute opioid overdoses are often required. More than 80% of overdoses are accidental and many are reversible through respiratory support and administration of an opioid antagonist such as naloxone.^6^^–^^9^

Naloxone is available in medical facilities in many countries and, since the 1990s, efforts have been made to provide non-medically trained people with the drug through take-home naloxone programmes.^8^^,^^10^ Such programmes are now included in the World Health Organization’s (WHO) recommended response to opioid overdose.^8^^,^^10^ Take-home naloxone programmes involve training lay people likely to witness an overdose, such as the friends or family of people at risk, in overdose recognition (e.g. signs such as cyanosis) and how to respond through, for example, rescue breathing and naloxone administration.^11^ The evidence shows that these programmes increase participants’ knowledge, confidence and skills in managing opioid overdoses.^11^^–^^13^ Moreover, they appear to be cost-effective and to reduce overdose deaths.^14^^–^^16^ Importantly, there is no evidence that take-home naloxone leads to riskier drug use behaviour.^17^ Although take-home naloxone is now used around the world, there are few publications from low- or middle-income countries, where little is known about opioid use or overdoses and where there may be limited access to emergency medical services.^18^^–^^20^ Studies of opioid overdose prevention have been carried out in Kyrgyzstan and Tajikistan and overdose prevention has been investigated in Kazakhstan as part of a broader evaluation of an intervention to reduce the risk of human immunodeficiency virus and hepatitis C virus infections.^21^ The take-home naloxone pilot programmes in Kyrgyzstan and Tajikistan involved only people who inject drugs: they were trained in overdose responses and given either vouchers for naloxone (Kyrgyzstan) or naloxone itself (Tajikistan). Subsequently, 83% (109/131) of programme participants who returned for additional naloxone in Kyrgyzstan and 30/59 (51%) in Tajikistan reported they had used naloxone at the last overdose witnessed.^20^ However, naloxone use by participants who did not request more was unknown. In Kazakhstan, the programme focused on couples, at least one of whom reported injecting heroin.^21^ All participants were trained in overdose responses and naloxone use and given vouchers for take-home naloxone. Although only 36% (148/414) of participants redeemed their vouchers, 71% (105/148) of those reported using naloxone on themselves or others, indicating that most would use the drug if available.^21^

In 2017, at the United Nations Commission on Narcotic Drugs meeting in Vienna, the United Nations Office on Drugs and Crime (UNODC) and WHO launched the Stop Overdose Safely (S-O-S) initiative within the framework of the UNODC–WHO Programme on Drug Dependence Treatment and Care.^22^^,^^23^ This initiative, which targets opioid overdose, was developed in response to recommendations of the 2016 Special Session of the United Nations General Assembly on the World Drug Problem and of the United Nations Commission on Narcotic Drugs resolution 55/7 (2012).^24^ The initiative’s aims are aligned with WHO’s guidelines on the community management of opioid overdose, which state that, “people likely to witness an opioid overdose should have access to naloxone and be instructed in its administration to enable them to use it for the emergency management of suspected opioid overdose.”^8^

As part of the S-O-S initiative, a training package was developed and implemented in a project conducted in Kazakhstan, Kyrgyzstan, Tajikistan and Ukraine – low-income to upper-middle-income countries that have widely varying policies and practices on drug law enforcement and treatment. The S-O-S training project in these countries began with stakeholder consultations and a review of drug policy, of the legal status of naloxone, and of any considerations affecting the use of take-home naloxone (Table 1).^27^ The final training model had three levels: (i) level-I trainers instructed level-II trainers in each country; (ii) level-II trainers instructed level-III training providers; and (iii) level-III training providers instructed potential opioid overdose witnesses. Specific training materials were developed for each level. Training of potential witnesses involved recognizing opioid overdose signs and symptoms, responding to overdoses, understanding naloxone and its use, and preventing future overdoses. Details of the overall theory of change and the programme logic are available in the data repository.^28^ The target was to train and distribute take-home naloxone to 4000 potential opioid overdose witnesses in each country.

**Table 1:** Country characteristics, take-home naloxone training project, Kazakhstan, Kyrgyzstan, Tajikistan and Ukraine, 2019–2020

Characteristic	Kazakhstan	Kyrgyzstan	Tajikistan	Ukraine
Estimated prevalence of opioid use,^25^ %	1.0	0.8	0.5	1.0
Estimated no. of people who inject drugs^26^	116 840	25 000	25 000	332 500
Opioid agonist treatment coverage for people who inject drugs,^26^ %	0.2	4.9	2.7	2.5
Naloxone availability^a^	•Not available on prescription •No take-home naloxone •Limited emergency service availability	•Available on prescription •Some take-home naloxone •Limited emergency service availability	•Available on prescription •Limited take-home naloxone •Some emergency service availability	•Some take-home naloxone •Emergency service availability •Available at some pharmacies for limited purchase
Naloxone carriage law^a^	Carrying syringes and naloxone is not an offence	Carrying syringes and naloxone is not an offence	Carrying syringes and naloxone is not an offence	Carrying syringes and naloxone is not an offence
Issues for lay responders to opioid overdoses^a^	•Limited legal protection •Prosecution is possible but unlikely for responding or even not responding	•First aid response mandated for trained professionals •No legislation for non-professionals	•Strong legal protections for responders in the case of “extreme need”	•All citizens expected to respond
Drug law^a^	•Drug purchase is a criminal offence •Criminal and civil penalties for use or possession	•Drug purchase is a criminal offence •Criminal or civil penalties for selling or possession, depending on quantity	•Drug purchase is a criminal offence •Criminal penalties for selling or possession	•Drug purchase is a criminal offence •Criminal and civil penalties possible for use or possession
Cities where the training project was implemented	Almaty	Bishkek, Sokuluk and Kant	Dushanbe and Khorugh	Kyiv

^a^ Data were obtained from legal reviews and assessments carried out in 2016.

During implementation of the training project, we carried out a prospective, observational cohort study to assess its impact. Here, we report on whether naloxone was used at witnessed opioid overdoses. Naloxone use at witnessed overdoses is a tangible and fundamental goal of take-home naloxone programmes that reflects both naloxone carriage by programme participants and whether training and naloxone carriage lead to its use at witnessed overdoses. We set an evaluation target of 90% of participants in the training project in Kazakhstan, Kyrgyzstan, Tajikistan and Ukraine using naloxone at witnessed opioid overdoses – a figure higher than previously observed in two of these countries.^20^^,^^21^

## Methods

**Fig. 1:**
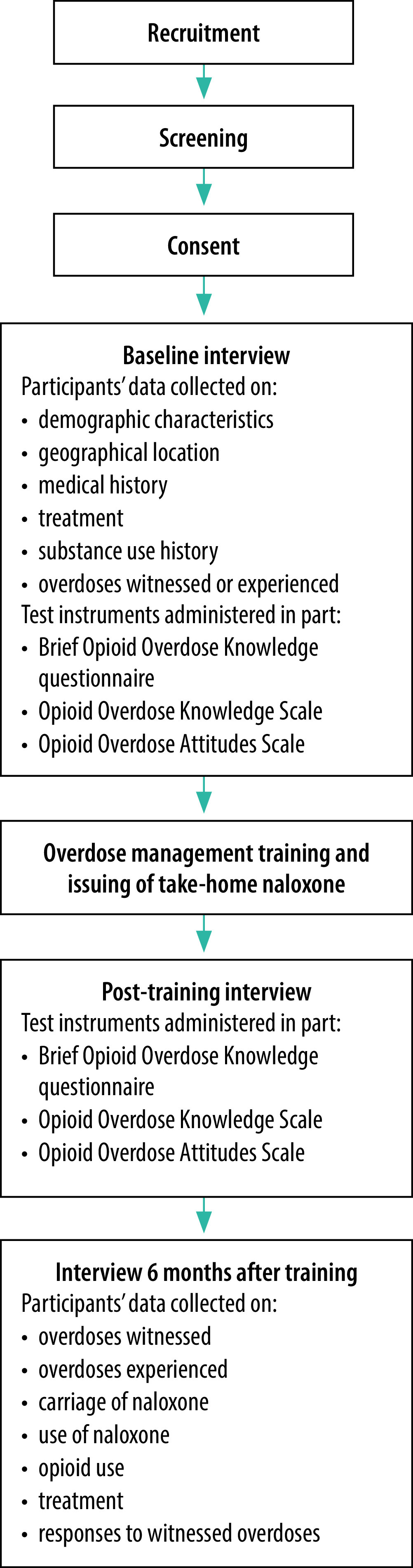
Flow chart showing cohort study schedule, take-home naloxone training study, Kazakhstan, Kyrgyzstan, Tajikistan and Ukraine, 2019–2020

We recruited and interviewed a sample of project participants in Kazakhstan, Kyrgyzstan, Tajikistan and Ukraine before, immediately after and 6 months after overdose management training (Fig. 1). Recruitment took place between July and October 2019 at all study sites and follow-up ended in April 2020.

The whole training project was advertised primarily by word of mouth and using recruitment flyers posted at locations frequented by people who used opioids or who were likely to witness an overdose, such as outreach and clinical services staff. For our cohort study, a convenience sample of trainees was recruited from within the project, also by word of mouth, until a target number was reached in each country. Eligible participants were people likely to witness an opioid overdose (e.g. those who used opioids, their family members or friends, and contact workers). Participants had to be: (i) aged 18 years or older; (ii) resident in the study city for 6 months or more; (iii) able to fluently speak and read the language of the study instruments (i.e. Russian); (iv) willing to provide written informed consent; (v) willing to undergo follow-up assessment at 6 months; and (vi) willing to provide contact details, including their name, residential address, home and mobile telephone numbers and social media details as well as the corresponding details of a friend or relative who would know their whereabouts if they could not be contacted directly.

The broad training project involved one-to-one or group-based training on the effects of opioids, on recognizing overdoses, and on responding in accordance with S-O-S manuals. After training, participants received small plastic boxes containing two safely wrapped, 400 µg ampoules of naloxone, two clean needles and syringes for intramuscular administration, disposable gloves, alcohol swabs, an instruction leaflet and a note of endorsement from the relevant authorities. Each naloxone kit cost 1.70 United States dollars (US$).

People who agreed to participate in the cohort study were informed about study procedures by trained staff. They were given an information sheet that described the study in detail, including its procedures and the possible risks and benefits of participation, after which written informed consent was obtained. Questionnaires were administered before, immediately after and 6 months after training (Fig. 1). Questionnaire responses were recorded on electronic devices programmed using Research Electronic Data Capture (REDCap) software (Vanderbilt University, Nashville, United States of America) or on hardcopy equivalents (details were subsequently entered onto REDCap forms). Data were uploaded directly onto the REDCap server at the Burnet Institute in Australia when a secure internet connection was available.

Participants in the cohort study were offered a cash reimbursement for their time, out-of-pocket expenses and transport costs at a local currency equivalent of 3–8 US$. Research assistants in each country attempted follow-ups after 6 months using the participants’ contact details and the details of nominated friends or relatives. Participants who could not be contacted and interviewed within 8 months were deemed lost to follow-up. Data were collected in person or by telephone. Participants could request additional naloxone kits at any time but data on naloxone use were collected formally only at follow-up. 

### Measures

At baseline (i.e. before training), demographic information, including age, sex, educational level, marital status, religious background, accommodation and employment, was collected. In addition, participants’ drug use history was assessed using the Drug Abuse Screening Test and their alcohol consumption in the previous 12 months was assessed using the Alcohol Use Disorders Identification Test for Consumption.^29^^,^^30^ Study-specific questions were designed to capture overdose history – both witnessed and experienced. Questions about behaviours engaged in when witnessing an overdose were drawn from the Opioid Overdose Knowledge Scale,^31^ a validated questionnaire that can be used to examine overdose responses. Questions about the participant’s knowledge of overdoses were taken from the Brief Opioid Overdose Knowledge questionnaire,^32^ a validated scale used to assess knowledge of overdoses among people who use prescribed opioids or who use opioids non-medically, and questions about attitudes towards, and perceived competence in responding to, overdoses were taken from the validated Opioid Overdose Attitudes Scale.^31^

Immediately after training, participants again answered questions from the Opioid Overdose Knowledge Scale, the Brief Opioid Overdose Knowledge questionnaire and the Opioid Overdose Attitudes Scale.

Six months after training, participants’ responses to witnessed overdoses were assessed using a modified version of the baseline questionnaire that included questions on the carriage of naloxone, witnessed overdoses and actions, overdoses experienced, opioid use, and treatment, and that was based partly on a questionnaire from a pilot trial of prison-based naloxone-on-release.^33^

### Analysis

The primary outcome of our study was naloxone use at witnessed overdoses reported at 6-month follow-up and the target for the proportion of respondents in each country who reported naloxone use was 90%. We calculated 95% confidence intervals (CIs) for this proportion, which allowed for a margin of 5% as a reasonable indicator of whether the target had been achieved. Assuming that 50% of opioid-consuming participants would witness an overdose each year and that 10% of participants who did not report consuming opioids would witness an overdose each quarter,^20^^,^^34^ it was estimated that roughly one third of study participants would witness an overdose during the follow-up period. Consequently, sample size calculations indicated that 408 participants were required in each country to achieve an estimated 138 witnessed overdoses by 6 months. Secondary outcomes related to programme implementation included: (i) the proportion of participants who still had the naloxone received at training; (ii) the proportion who told other people that they had access to naloxone; and (iii) the proportion who had carried naloxone during the previous 3 days. We also asked about the survival of the person whose overdose was witnessed. The characteristics of the study sample in each country are reported using descriptive statistics. Given the diversity of study participants between countries, no analysis of outcomes by participants’ characteristics was undertaken.

### Ethical approval

Ethical approval of the study protocol was obtained from the WHO Ethics Review Committee (ERC.0003090, 13 November 2018) and from local ethics committees in Kazakhstan (Medical Faculty, Higher School of Public Health, Al-Farabi Kazakh National University; N 1236, 31 July 2018), Kyrgyzstan (Bioethical Committee, Republican Center of Narcology, Kyrgyz Republic Ministry of Health; N 952, 6 September 2018), Tajikistan (Biomedical Committee, Academy of Medical Science, Republic of Tajikistan Ministry of Health and Social Protection; N 92, 14 August 2018) and Ukraine (Institutional Review Board, Ukrainian Institute of Public Health Policy; N 29/IRB, 1 August 2018).

## Results

Table 2 shows the sociodemographic characteristics of the study participants in each country. The target sample size of 408 was achieved in all countries except Ukraine, which had 400 participants. Across the countries, the participants’ mean age ranged from 38 to 42 years. The majority were employed and few reported homelessness in the previous 6 months. However, all other characteristics differed between countries: the reported educational levels were higher in Tajikistan and Ukraine; the proportion of women was higher in Kazakhstan and Kyrgyzstan; and the proportion of married participants was higher in Kyrgyzstan and Tajikistan. Over 80% of participants were retained in the study at 6 months, ranging from 82% (341/417) in Kazakhstan to 86% (342/400) in Ukraine.

**Table 2:** Sociodemographic characteristics of participants, take-home naloxone training study, Kazakhstan, Kyrgyzstan, Tajikistan and Ukraine, 2019–2020

Characteristic	No. (%)^a^			
	Kazakhstan (*n* = 417)	Kyrgyzstan (*n* = 412)	Tajikistan (*n* = 417)	Ukraine (*n* = 400)
**Mean age in years (range)**	42 (19–65)	40 (19–68)	41 (19–70)	38 (18–60)
**Sex**				
Female	127 (31)	146 (35)	78 (19)	85^b^ (21)
Male	290 (69)	266 (65)	339 (81)	314^b^ (79)
**Relationship**				
Married	88 (21)	158 (38)	239 (57)	84 (21)
Other	329 (79)	254 (62)	178 (43)	316 (79)
**Religion**				
Muslim	88 (21)	139 (34)	397 (95)	7 (2)
Christian	263 (63)	199 (48)	≤ 5 (< 2)	306 (77)
Other	66 (16)	74 (18)	< 5 (< 2)	87 (22)
**Education**				
Did not complete high school	171 (41)	176 (43)	54 (13)	55 (14)
High school education at least	246 (59)	236 (57)	363 (87)	345 (86)
**Employment**				
Employed full or part time	240 (58)	240 (58)	253 (61)	232 (58)
Not employed	177 (43)	172 (42)	164 (39)	168 (42)
**Housing**				
Home owned or rented	191 (46)	231 (56)	228 (55)	266 (67)
Other	226 (54)	181 (44)	189 (45)	134 (34)
**Homeless in the past 6 months**				
Yes	60 (14)^c^	30 (7)	48 (12)	27 (7)^c^
No	356 (86)^c^	382 (93)	369 (88)	372 (93)^c^

^a^ All values in the table represent absolute numbers and percentages unless otherwise stated.

^b^ The sex of one participant was unknown.

^c^ The homelessness status of one participant was unknown.

Note: Inconsistencies arise in some values due to rounding.

The percentage of participants who reported witnessing an overdose between baseline and follow-up at 6 months was 20% (71/356) in Tajikistan, 33% (113/349) in Kyrgyzstan, 37% (125/342) in Ukraine and 50% (170/341) in Kazakhstan. The percentage of overdose witnesses who reported using naloxone at their most recently witnessed overdose was 89% (152/170) in Kazakhstan, 89% (101/113) in Kyrgyzstan and 100% (71/71) in Tajikistan (Table 3). However, the figures for Kyrgyzstan and Tajikistan should be treated with caution because the number of participants who witnessed an overdose was below that expected (i.e. 138 per country), meaning the precision of the estimates was lower than expected. The percentage of participants in Ukraine who reported using naloxone at their most recently witnessed overdose was 82% (103/125, 95% CI: 75–88), which fell just below the target of 90%. In almost all reported overdoses in which naloxone was used, the overdose victim survived (Table 3). The percentage of participants at 6-month follow-up who still had one or both ampoules of the naloxone they received at enrolment was 89% (316/356) in Tajikistan, 72% (245/342) in Ukraine, 53% (184/349) in Kyrgyzstan and 45% (154/341) in Kazakhstan (Table 4). Over 80% of participants reported they had told others they had naloxone (Table 4). The percentage who reported carrying naloxone in the 3 days before the 6-month follow-up varied substantially from 17% (58/342) in Ukraine to 89% (317/356) in Tajikistan (Table 4).

**Table 3:** Naloxone use and overdose victim survival reported at 6-month follow-up, take-home naloxone training study, Kazakhstan, Kyrgyzstan, Tajikistan and Ukraine, 2019–2020

Outcome	Kazakhstan	Kyrgyzstan	Tajikistan	Ukraine
No. participants followed up at 6 months who witnessed an opioid overdose	170	113	71	125
No. participants who reported administering naloxone at their most recently witnessed overdose^a^	152	101	71	103
Percentage of participants who reported administering naloxone at their most recently witnessed overdose, % (95% CI)	89 (84–93)	89 (82–94)	100 (NA)	82 (75–88)
No. overdose witnesses who used naloxone and reported the victim’s survival status	151^b^	101	71	102^b^
No. (%) overdose victims reported as surviving	149 (99)	101 (100)	70 (99)	98 (96)

CI: confidence interval; NA: not applicable.

^a^ Overdoses were witnessed at any time during the 6-month follow-up period.

^b^ Information on survival was not available for one victim.

**Table 4:** Naloxone availability at 6-month follow-up, take-home naloxone training study, Kazakhstan, Kyrgyzstan, Tajikistan and Ukraine, 2019–2020

Outcome	Kazakhstan	Kyrgyzstan	Tajikistan	Ukraine
No. participants followed up after 6 months	341	349	356	342
No. (%) of those followed up who reported still having the naloxone issued at project enrolment	154 (45)	184 (53)	316 (89)	245 (72)
No. (%) of those followed up who told others about carrying naloxone	310 (91)	345 (99)	356 (100)	288 (84)
No. (%) of those followed up who carried naloxone in the 3 days before follow-up	85 (25)^a^	49 (14)	315 (89)^a^	58 (17)

^a^ Information on naloxone carriage was not available for one participant.

## Discussion

Our prospective cohort study aimed to determine whether 90% of participants in an S-O-S training project in Kazakhstan, Kyrgyzstan, Tajikistan and Ukraine used naloxone when they witnessed an opioid overdose. The target of 90% was achieved in three of the four countries (and very close to being achieved in the fourth) and naloxone use was greater than observed previously.^20^^,^^21^ In almost all reported instances of naloxone use, the recipient survived. Our findings demonstrate that the project resulted in use of project-provided naloxone in a variety of settings.

One WHO–UNODC target for take-home naloxone is that 90% of trained potential witnesses should carry the drug or have it available for use.^23^ Although our study was not specifically designed to assess carriage of naloxone, we found low reported rates of naloxone carriage, which were below the UNODC–WHO target and similar to previously observed rates.^35^ However, this did not lead to a low rate of naloxone use at witnessed overdoses. We assessed naloxone carriage using a single question drawn from the N-ALIVE study;^33^^,^^36^ we asked only whether naloxone had been carried in the previous 3 days, not whether programme-issued naloxone was available for use. Our findings suggest that this question was inappropriate for characterizing naloxone carriage as naloxone was clearly available when needed. Indeed, other information collected by the project team suggest that naloxone was available in locations where people may witness an overdose and was stored there rather than carried. Future work should use a measure of naloxone access that can better capture the availability of naloxone for responding to opioid overdoses. 

Our study was a single-arm observational study appropriate for characterizing naloxone use by people provided with take-home naloxone in the training project. The study involved a large number of potential overdose witnesses in four diverse countries and had a retention rate over 80% at 6 months. The study did not seek to examine the effectiveness of the project in preventing overdose fatalities, which would have required a different study design. Nevertheless, although it is unknown how many overdoses would have proved fatal had naloxone not been used, participants using naloxone likely reversed potentially fatal overdoses. We base this conclusion on studies showing only around one quarter of overdose witnesses report calling an ambulance in Central Asian countries,^20^ where emergency medical responses may be unavailable and fatal outcomes may, therefore, be more likely. In contrast, up to 78% of witnesses call ambulances in more-developed countries.^37^

Our study findings suggest that implementing an S-O-S training project in low- and middle-income countries is feasible and can lead to naloxone use at witnessed overdoses. However, project implementation required substantial advance research, consultation and programme development to overcome numerous challenges. Analyses of local policing practices, for example, showed that awareness of opioid overdoses was low in most countries. As a result, project implementers designed and conducted first aid training for police officers in Kyrgyzstan and specific training in overdose responses for some police officers in Ukraine. Concerns about the police’s attitude to naloxone carriage led to the inclusion of a note in naloxone kits indicating that the kits were endorsed by government authorities, even though no country had legislative barriers against the carriage of either naloxone or needles and syringes. In addition, the availability of naloxone in ambulance services varied across countries. These situational factors and other barriers to implementation need to be considered in future S-O-S training projects. Finally, plans for sustaining the project must be made before implementation. In our study countries, take-home naloxone programmes continue but resources have not been allocated to allow their expansion.

Our study was limited by the use of a convenience sample of participants and by reliance on self-report questionnaires to assess outcomes at 6 months. Self-report has proved reliable in studies involving people who use drugs,^38^ but it is not known whether recall or other biases influenced responses to questions about overdoses in our study. Although our sampling strategy means our findings are not directly generalizable to other settings, it is encouraging that similar findings have been observed in diverse low- and middle-income countries. Finally, the expected number of study participants who reported witnessing an overdose was reached in only one of the four study countries, meaning that the precision of the estimated margin used to assess the naloxone use target in the remaining three countries was lower than expected.

In conclusion, our study showed that the implementation of a WHO–UNODC S-O-S training project can result in the successful use of take-home naloxone at around 90% of witnessed opioid overdoses in low- to middle-income countries.
